# What About Affected Family Members of Disordered Gamblers During the COVID-19 Pandemic? A Study in Italy During the Lockdown Restrictions

**DOI:** 10.3389/fpsyg.2022.801835

**Published:** 2022-04-26

**Authors:** Maria Anna Donati, Daniela Capitanucci, Carola Beccari, Roberta Smaniotto, Eleonora Quadrelli, Alfredo Casini, Caterina Primi

**Affiliations:** ^1^Section of Psychology, Department of Neuroscience, Psychology, Drug, and Child's Health, University of Florence, Florence, Italy; ^2^Association Azzardo e Nuove Dipendenze [AND (Gambling and New Addictions)], Gallarate, Italy; ^3^Mental Health and Addiction Department of La Spezia, La Spezia, Italy

**Keywords:** gambling disorder, family affected members, COVID-19 lockdown, telephonic interview, psychological state

## Abstract

Although some studies have been conducted on gambling behaviour in the general population or in clinical samples during the COVID-19 pandemic, less attention has been focused on Affected Family Members (AFMs) of disordered gamblers. To fill this gap, this study investigated the psychological state of disordered gamblers' AFMs during the COVID-19 lockdown in Italy, the European country first affected by the virus and with the largest gambling market. We were interested in understanding if the unavailability of most land-based gambling offerings during the lockdown created a sense of relief in AFMs. We also compared the quality of family relationships and emotional state during the lockdown of AFMs with those of their relative with Gambling Disorder (GD). Participants were 53 disordered gamblers' AFMs (77% female; mean age = 50.28). For 42 out of the 53 AFMs, we also recruited their relative with GD (86% male; mean age = 48.98). A semi-structured telephone interview was developed. Although AFMs reported a general interruption of the relative's gambling behaviour and a general sense of relief for the closure of gambling activities, accompanied by the perception of good family relationships, AFMs still felt tired, worried, and apprehensive and perceived more fear, stress, and anxiety than before the lockdown. A large proportion of AFMs engaged in potentially addictive behaviours, especially TV and mobile phone and Internet use, which increased in frequency compared to before the pandemic. They still perceived some gambling-related problematic behaviours from their relative and put in place coping strategies to manage the gambling problem. Even if they had a general positive orientation towards the future, they experienced fear when thinking about the reopening of gambling opportunities at the end of the lockdown. Compared to their relatives with GD, AFMs appeared more prone to perceiving a general negative state and a worsening of it from before the lockdown. Overall, this study shows that disordered gambling's AFMs can be considered as a particularly at-risk group who deserves focused clinical attention even during gambling closures related to pandemic lockdown.

## Introduction

In the last 20 years, gambling activity has reached unprecedented levels worldwide. Despite the small extent of its territory, Italy contains the largest gambling market in Europe and the fifth largest in the world after the United States, Japan, China, and Macau (Global Betting and Gaming Consultants, 2019). In addition, 3% of the population, both adult and underage, have lost control over their gambling behaviour and can be considered problematic gamblers (Mastrobattista et al., 2019). In detail, spending (Agenzia Dogane e Monopoli; ADM, 2020, 2021), participation, prevalence of gambling disorder (GD) rates (Calado and Griffiths, 2016; Cavalera et al., 2018), and gambling related harm (Langham et al., 2016) have increased both nationally and internationally due to a strong increase of gambling accessibility (Abbott, 2020; Fiasco, 2020). Indeed, although gambling can be considered a recreational activity, numerous negative implications have been highlighted, including a variety of individual and societal harms ranging from ill health and financial issues, to crime, societal costs, and social inequalities (Jeanrenaud et al., 2012; Costes et al., 2014; Kohler, 2014; Browne et al., 2017a,b).

Gambling researchers have long argued that the increased availability and accessibility of gambling contributes to increasing the prevalence of problem gambling. The links between accessibility to gambling, gambling behaviour, and problem gambling have been examined in several studies (e.g., Marshall et al., 2004; Wheeler et al., 2006), all of which have found evidence of significant and positive relationships. Some longitudinal studies have shown both immediate and mid-term increases (Ladouceur et al., 1999; Jacques et al., 2000) and subsequent stabilisation (Jacques and Ladouceur, 2006) of participation, GD prevalence rates, and other related indices. Exposure and adaptation models provide competing perspectives of the environmental influence on the development of addictive disorders. Exposure theory suggests that the presence of environmental toxins (e.g., casinos) increases the likelihood of related disease (e.g., gambling-related disorders). Adaptation theory proposes that new environmental toxins initially increase adverse reactions. Subsequently, symptoms diminish as individuals adapt to such toxins and acquire resistance (Shaffer et al., 2004). Storer et al. (2009) found that access and adaptation very likely work simultaneously.

The role of environmental-contextual factors, which can act as risk or protective variables, became of particular interest when the World Health Organization (WHO) officially declared a global pandemic due COVID-19 on 11 March 2020 (Cucinotta and Vanelli, 2020). After which, people's life context changed dramatically on many levels, including gambling offerings and habits. Italy was the first country in Europe to be hit by the virus in late January 2020. To face the new epidemic emergency, the Italian Government declared a rigid “lockdown” period. From 9 March to 4 May 2020, a national-level stay-at-home order was given, and strict restrictions were enforced. Individual freedom of movement was limited and only indispensable activities were allowed throughout the entire country (Presidenza del Consiglio dei Ministri, 2020). Among the public health measures taken, all unnecessary activities were shut down. This resulted in the general unavailability of most land-based gambling offerings, particularly Electronic Gambling Machines (the most prevalent gambling activity practised by Italian patients with GD) which were banned from 9 March to 19 June 2020 (ADM, 2020). The suspension of most of sporting events worldwide led to the cessation of related betting, both land-based and online. Due to the unavailability of most chance-based gambling games, people lived for about three months without having the opportunity to gamble easily. This scenario gradually spread to the rest of Europe, creating a ‘totally different context' for gambling compared to only a few months before the pandemic broke out (Marshall, 2009).

This situation, which might even be considered “the world's biggest psychological experiment” (Hoof, 2020), challenged several researchers to study the behavioural and psychological effects connected with the decreased availability of chance-based gambling games and, with the reduced exposure to land-based gambling offerings on the general population, gamblers, and disordered gamblers. A fair body of research on the topic of gambling and COVID-19 has been developed, and systematic reviews have already been published (Brodeur et al., 2021; Hodgins and Stevens, 2021). Two main research trajectories have been explored. The vast majority investigated the general population of gamblers (e.g., trends in gambling consumption before/during and after the lockdown; switch from land-based to online gambling, well-being vs. malaise, access to services). Contrary to expectations, which assumed a shift towards online gambling, preliminary evidence (Gunstone et al., 2020; Håkansson, 2020) suggested that gambling behaviour often either decreased or stayed the same for most gamblers during the pandemic. For the minority who showed increased gambling behaviour, however, there was a frequent association with problem gambling (Brodeur et al., 2021). The longer-term implications of both the reduction in overall gambling and the increase in some vulnerable groups are unclear and require assessment in subsequent follow-up studies (Hodgins and Stevens, 2021). Two studies, both performed in Italy, have investigated samples of disordered gamblers in treatment facilities (outpatients, living at home during lockdown, or inpatients in residential programmes) throughout the whole lockdown phase (Martinotti et al., 2020; Donati et al., 2021). Martinotti et al. (2020) evaluated the impact of the COVID-19 pandemic and the relative containment measures on small samples of patients suffering from substance use disorders (SUDs) and/or GD. They found no substantial psychopathological difference between the SUD and GD sample and the general population. The presence of a moderate psychopathological burden correlated to poor quality of life and low craving scores represented their main outcomes. Their main finding was that gambling craving was low when the accessibility of gambling opportunities decreased during lockdown. Donati et al. (2021) interviewed 135 problem gamblers in treatment and found that most of them achieved a significant improvement in their quality of life, with less gambling behaviour, fewer GD symptoms, and lower craving. No shift towards online gambling and a very limited shift towards other potential addictive and excessive behaviours occurred. The longer the treatment, the more monitoring was present and the better were the results in terms of the reduction of symptoms.

Scant attention has been focused on studying what occurred among the families of people in treatment for GD. GD affects not only gamblers but also their families' resources, relationships, and health. Following the estimations by the WHO that about 100 million people are affected by someone else's drug or alcohol addiction (Tepperman and Mills, 2009), it has been hypothesised that equally important numbers relate to people experiencing a relative's gambling addiction (Copello et al., 2010; Kourgiantakis et al., 2013). Several studies have estimated that between six and 10 persons in a gambler's social network are directly affected by that person's gambling problem (Goodwin et al., 2017). In Finland, 21% of concerned significant others experienced harm due to the gambling of others (Marionneau and Jarvinen-Tassopoulos, 2021). Broadly speaking, “affected others” are people who know someone who has had a problem with gambling (either currently or in their past) and feel that they have personally experienced negative effects because of that person's gambling behaviour. Affected family members (AFMs) could include family members, friends, and work colleagues, amongst others, with the negative effects ranging from financial to emotional or practical impacts (Gunstone et al., 2020). According to Browne et al. (2017a,b), both gamblers and those around them are affected by harms including reductions in health (morbidity and mortality), emotional or psychological distress, financial harm, reduced performance at work or education, relationship disruption, conflict or breakdown, and criminal activity. Holmes et al. (2020) underlined how although the whole population is affected by the COVID-19 pandemic, specific sections of the population have experienced it differently. Children, young people, and families might also be affected by exposure to gambling, substance misuse, domestic violence and child maltreatment, accommodation issues and overcrowding, parental employment and change, and disruption of social networks.

Bischof et al. (2020) noted that although the AFMs of individuals suffering from addiction generally show elevated levels of stress and strain, AFMs have not been adequately addressed by research and care in the lockdown period. As a result, reliable data on the effects of the social restrictions caused by the COVID-19 pandemic on burden and care for AFMs are missing. Surveying international experts and German treatment providers' opinions, Bischof and colleagues found the expected increased strain to be due to the effects of the pandemic, which was linked to the increased risky behaviour of the addicted family member and an increase of interpersonal conflict. However, they also reported some possible positive effects on subgroups of AFMs. Overall, treatment providers noted a decline in care supply for relatives and a trend for reduced demand for help by AFMs. In line with these conclusions, Marionneau and Jarvinen-Tassopoulos (2021) found that in parallel to the decreased availability of gambling opportunities, COVID-19 lockdowns and social distancing regulations have also affected treatment and help services for gamblers and AFMs. During spring 2020, some services were closed, while others moved online. Their study also investigated the experiences and views of 97 AFMs of gamblers on treatment and help services in Finland and collected their suggestions for how services and prevention should be better organised during and after COVID-19. The respondents were familiar with the treatment and help services and had valuable insights into the question. Because of a different research goal (they focused on the organisational responses of the care services and not on the psychosocial needs expressed by the patients and AFMs), they unfortunately excluded questions related to the impacts of the COVID-19 pandemic on family relationships, well-being, or financial circumstances. Finally, in Quebec, Brodeur (2021) is conducting a two-year research project to assess the impact of the COVID-19 crisis on gambling habits. The first quantitative-qualitative phase has already been carried out and had objectives that are specifically aimed at gamblers. The second qualitative phase will be carried out between autumn 2021 and spring 2022. It will include interviews with 30 gamblers and 30 family members that aim to understand their experiences and moods during the pandemic. As far as we know, no other studies exist on the topic.

The above cited data attest that AFMs represent a neglected group of suffering people in need of assessment and research studies. In particular, AFMs experience high levels of burden (Orford et al., 2017). Specific characteristics that distinguish disordered gamblers' AFMs make them a particularly vulnerable population. Indeed, according to the Stress-Strain-Coping-Support Model (Orford et al., 2010), having a close relative with substance or behavioural misuse constitutes a form of stressful life circumstances that are often longstanding, which puts AFMs at risk of experiencing strain in the form of physical and/or psychological ill-health. Coping and social support are the two other central building blocks of the model. AFMs are viewed as ordinary people faced with the task of coping with such stressful life circumstances. It is an assumption of the model that, as difficult as the coping task is, family members need not be powerless in maintaining their own health and helping their relatives. Good quality social support in the form of emotional support, good information, and material help is an invaluable resource for AFMs, supporting their coping efforts and contributing positively to their health and increasing the positive social support available from professional sources. The peculiarity of family members of gamblers is that well before the pandemic, they had already been living for a long time in a context characterised by stress, strain, and, often, poor social support, forced to search for coping strategies more appropriate to different situations characterised by fear, uncertainty, and changeability. These circumstances, sometimes even of a practical nature, such as economic or work difficulties, were overlapped and exacerbated by the onset of COVID-19. The living conditions normally experienced by family members of gamblers, i.e., stress, strain, need to readjust coping strategies and lack of support, predisposed them to adverse conditions that were further reinforced during the pandemic lockdown. Social isolation due to COVID-19 enhanced the previous social isolation due to being family members of a disordered gambler, worsening their already compromised health and mental and emotional state. It could be assumed that their situation of previous vulnerability was amplified by pandemic restrictions and threats, given the symptoms found in the general population. Recently, just before the COVID-19 outbreak, Klevan et al. (2019), using in-depth interviews, acquired a thorough understanding of gamblers' partners' experiences of everyday life, relations, and parenting. They reconfirmed that living with a partner struggling with a gambling problem severely affects numerous aspects of everyday life, including relations and parenting. Experiences of loss and loneliness across a span of issues connected to family life were striking. Partners of persons with gambling problems found themselves inhibited by the situation. They faced restrictions because of concrete issues, like poor finances, increased responsibility for taking care of practicalities in the family, and a lack of support on these issues from both their partner and the service system. Furthermore, they also experienced emotional loneliness in the relationship with their partner through the shame and stigma connected to their gambling problem and choosing to stay with a problem gambler.

The hypothesis that the difficult situation of disordered gamblers' AFMs could be exacerbated during the COVID-19 lockdown is supported by research studies that highlight the negative impact on mental health and well-being during pandemics in the general population. Particularly, psychological distress, such as increases in anxiety, depression, stress, anger, fear, sleep, and eating disorders has been found all over the world (e.g., Dubey et al., 2020; Mazza et al., 2020; Rossi et al., 2020). Furthermore, it has been seen that the COVID-19 lockdown presented significant economic, psychosocial, and physical risks (e.g., domestic violence) to the well-being of women and their families, particularly across the most socioeconomically deprived strata of the general population (Hamadani et al., 2020). These considerations allow us to consider disordered gamblers' AFMs as a specific vulnerable/at-risk group in general and particularly during the COVID-19 pandemic. Researchers should thus have a specific interest in deepening AFMs' psychological, emotional, and relational situation during the COVID-19 pandemic.

Following these premises, the final goal of the current study was to investigate the psychological state of disordered gamblers' AFMs during the COVID-19 lockdown in Italy. We were interested in understanding if the unavailability of most land-based gambling offerings during the lockdown created relief for AFMs. Moreover, to better understand the specificity of this target group, we also compared AFMs to their relative with GD in relation to the quality of family relationships and emotional state during the lockdown. In detail, we aimed to describe AFMs' life conditions during the lockdown, with particular attention to their emotional state, psychological distress, involvement in potentially addictive behaviours, and their personal perceptions of changes in their life because of the lockdown, and also their personal relationship with the COVID-19 disease and related restrictions. We also investigated AFMs' perceptions of their relative's gambling behaviour, their gambling-related emotions and feelings, their coping strategies to face their relative's gambling addiction, and their expectations for the future. Finally, we compared AFMs to their disordered gambler relatives in terms of perceived quality of family relationships and emotional state.

Lockdown restrictions made it impossible to assess AFMs and the patients in person, so we developed a semi-structured telephone interview to be conducted by the health-care professional in charge of the gambler patients or/and the AFMs themselves before the lockdown as a monitoring tool. We expected to find a low involvement in gambling for patients with GD in treatment during the lockdown (Donati et al., 2021) due to the predominant closure of land-based gambling activities. Given that having a close relative with a behavioural addiction represents a chronic stressful situation (Orford et al., 2010) and that AFMs of individuals suffering from addiction generally show elevated levels of stress and strain (Bischof et al., 2020), we hypothesised that we would find a general emotional state of stress, fear, and tension among AFMs, along with the psychological consequences of the hard situation due to lockdown restrictions, as generally highlighted (e.g. Brooks et al., 2020; Dubey et al., 2020; Mazza et al., 2020; Rossi et al., 2020). Finally, we predicted that we would obtain different types of responses from the AFMs and their matched gambler relative. Given the above cited chronic stress, we expected to find a lower prevalence of a general positive state and a higher prevalence of its worsening subjective perception during the lockdown among the AFMs.

## Materials and Methods

### Participants

Participants were 53 AFMs of disordered gamblers (77% females; mean age = 50.28; *SD* = 13.34; range: 27–77 years) recruited in the north of Italy by the health services at which the disordered gamblers were in treatment. The majority of the participants were contacted through the National Health Drugs Services (Ser.D.) and through the private non-profit association Azzardo e Nuove Dipendenze [AND (Gambling and New Addictions)] in Gallarate (Lombardy) (Table 1). Concerning educational level, the majority of the AFMs had a high school diploma and half of the sample were employed; most were married, and in terms of kinship with the gambler, the majority were the spouse/cohabitant.

**Table 1:** Affected family members' educational level, occupational status, marital status, kindship with the gambler and gambler's recruitment service.

**Educational level**	**% (*n*)**
Elementary school	6 (3)
Middle school diploma	30 (16)
High school diploma	47 (25)
University degree	17 (9)
Occupational status	
Employee	50 (27)
Self-employee	14 (7)
Retired	12 (6)
Housewife	15 (8)
Students	2 (1)
Unemployed	8 (4)
Marital status	
Married	62 (34)
Cohabiting	17 (9)
Single	8 (4)
Separate/Divorced	9 (5)
Widowed	2 (1)
Kindship with gambler	
Spouse/Cohabitant	57 (30)
Parents	19 (11)
Sibling	11 (6)
Son/Daughter	11 (6)
Gambler recruitment service	
Ser.D. of La Spezia	74 (39)
Ser.D. of Rovigo	11 (6)
AND, Varese	15 (8)

For 42 out of the 53 AFMs, we also recruited their relative in treatment for GD (86% males; mean age = 48.98; *SD* = 13.62; range: 28–73 years) (Table 2). They were interviewed by their reference operator within the services. Half of the gamblers were in the middle of the treatment for GD, and half of them were employed. Almost all were regular gamblers—that is, they gambled weekly or daily (Welte et al., 2009), and most of them used slot machines for gambling. According to the *South Oaks Gambling Screen* (SOGS; Lesieur and Blume, 1987; Italian version: Guerreschi and Gander, 2000), they were all classifiable as *problem gamblers* (*M* = 11.62; *SD* = 3.72, range: 6.00–16.00).

**Table 2:** Gamblers' occupational status, kindship with the affected family member, recruitment service, level of treatment and most practised gambling activities.

**Occupational status**	**% (*n*)**
Employee	50 (21)
Self-employee	19 (8)
Retired	21 (9)
Unemployed	10 (4)
Recruitment service	
Ser.D. of La Spezia	81 (34)
AND, Varese	19 (8)
Level of treatment	
In the middle of the treatment	50 (21)
Monitored	36 (15)
Pre-dismissal stage	12 (5)
Discharged	2 (1)
Most practised gambling activity^*^	
Slot Machines	82 (33)
Bets on sports events	6 (3)
Instant scratch cards	4 (2)
Online gambling	4 (2)
Bets on the stock market	2 (1)
Lotteries	2 (1)

* *The answer categories for this question are not mutually exclusive*.

### Procedure and Measures

#### AFMs

A semi-structured telephone interview was developed and conducted by the healthcare professionals (see Supplementary Material). Data were collected during the lockdown period due to the COVID-19 pandemic, specifically from 9 May 2020 to 18 June 2020. Participants completed the interview only after having understood the information sheet and having given their informed consent. The interviews lasted about 40 min, and participants were asked to respond with reference to the lockdown period.

The telephone interview was divided in four sections. The first section focused on the participants' life conditions and emotional state during the lockdown. The symptoms of psychological distress during lockdown were also investigated through the *Symptom Rating Test* (SRT; Kellner and Sheffield, 1973; Italian version: Fava et al., 1983). The SRT consists of 30 Likert-type items, ranging from 0 (*never*) to 2 (*often*), which assess the extent to which family members have experienced the described physical and psychological symptoms during the last month. An example item is “*Feeling tired or without energy*.” The occurrence and frequency of potentially addictive behaviours during the lockdown period were also investigated. For symptoms of psychological distress and potential addictive behaviours, in the scoring phase, options “*sometimes*” and “*often*” were collapsed to obtain an affirmative or negative answer with respect to having experienced the symptoms and having engaged in the described behaviours during the last month. The participants' perceptions about the changes from the pre-lockdown period to the current period concerning the relationships with family members, general emotional state, and symptoms of psychological distress experienced were also queried.

In the second section, questions centred on personal experience with the COVID-19 disease and attitude towards the national restrictions during the lockdown. In the third section, the gambling behaviour of the relative in treatment for GD, situations that occurred in the family because of the relative who gambles, the coping strategies used to manage their relative's gambling behaviour, and feelings with respect to gambling were investigated. In the scoring phase, the options “*once/twice*,” “*sometimes*,” and “*often*” were collapsed to obtain an affirmative or negative answer on the occurrence of the situations described. In the same way, for the coping strategy used to manage their relative's gambling behaviour, in the scoring phase options, “*once/twice*,” “*sometimes*,” and “*often*” were collapsed to obtain an affirmative or negative answer on the use of the coping strategies described. The participants' perceptions about changes from the pre-lockdown period to the current period were also investigated regarding gamblers' behaviour and the coping strategies used to manage their addicted relative's gambling behaviour. The fourth section focused on expectations regarding the future, with specific reference to feelings about the closure of gambling opportunities due to COVID-19 and the future easing of restrictive measures.

#### Relatives In-treatment for GD

A semi-structured telephone interview conducted by healthcare professionals was also developed for the AFMs' relative in treatment for GD. Data were collected from 7 April 2020 to 28 May 2020. Participants completed the interview after having given their informed consent. The interviews lasted ~40 min. During the telephone interview, the patients' life conditions (e.g., family relationships) and emotional state during the lockdown were investigated. The addicted patients' feelings towards gambling were also queried, along with participants' perceptions about changes in relationships with family members and general emotional state from the pre-lockdown period to the current period. These dimensions were assessed through equivalent/parallel questions with respect those used for the AFMs (see Supplementary Material).

Gambling frequency during the lockdown and gambling severity were investigated through the SOGS, the most widely used measurement instrument for gambling problem severity classification across the health services involved in the study in accordance with practises in the National Health Drugs Services in Italy (Capitanucci and Carlevaro, 2004). The SOGS is a 20-item questionnaire based on the DSM Third *Edition* (DMS-III; American Psychiatric Association, 1980) criteria for gambling problem. It is a widely used screening instrument for gambling problem and shows good reliability and validity in community and clinical samples (Stinchfield, 2002; Petry, 2005). For the purposes of this study, we omitted the non-scored items 2 and 3 that investigate the largest amount of money ever gambled with on any one day and parents' gambling problems, respectively. We also modified the original time frame by referring to the last month. The first item of the SOGS investigated the frequency of gambling (not at all = 0, less than once a week = 1, once a week or more = 2) in ten activities including playing cards for money, betting on horses, dogs or other animals, sport bets, dice games for money, casino, betting on traditional/instant lotteries, bingo, stock and/or commodities market bets, slot machines, poker machines or other gambling machines, and games of skill for money. To obtain further information about gambling frequency, taking into account the specificity of the lockdown period in the closure of legalised gambling venues, we also included online games for money and private bets with friends and family members.

## Results

### AFMs

#### Life Conditions During Lockdown

During lockdown, the AFMs of disordered gamblers generally lived in medium-size houses (*M* = 93.96, *SD* = 27.96, range: 42–230 m^2^), with terraces as the prevalent open spaces, and, on average, with about other two persons in the house (*M* = 2.06, *SD* = 1.05, range: 0–5 persons). Among those who continued to work (34%; *n* = 18), the majority (72%) went to the workplace, while 28% continued to work via “smart working,” an organisational model of work developed during the COVID-19 pandemic in which workers were able to work outside their workplace and with a flexible time schedule thanks to the use of technology (Angelici and Profeta, 2020). The relationships with the cohabitants were rated as quite good (*M* = 7.83, *SD* = 1.55, range: 4–10) and were judged as predominantly improved, generally due to greater family sharing. About their job, the majority of the participants who were employed still worked during the lockdown and continued to work traditionally. Taking care of the house was the prevalent activity done while at home. For general emotional state, participants' responses were classified in different categories based on the reported words. Responses in which terms such as “*well, better, calm, serene, happy*” appeared and were treated as responses reflecting a general positive state, while responses with terms such as “*tired, bored, depressed, sad, worried*” were treated as responses reflecting a general negative state. In about a fifth of the sample, a general positive state was found, while the remainder of the sample reported a general negative state because of fear, anxiety, and mood swings. One participant made an explicit reference to gambling, reporting concern for the reopening of slot venues. Although many respondents reported a general positive emotional state, most of the participants described their emotional state as worsened with respect to before the lockdown (Table 3).

**Table 3:** Affected family members' life conditions and characteristics during lockdown.

	**% (*n*)**
Open spaces in the house^*^	13 (7)
Terrace(s)	59 (31)
Garden(s)	47 (25)
Vegetable garden(s)	9 (5)
Number of other persons in the house	
No one	2 (1)
One	32 (17)
Two	36 (19)
More than two	31 (16)
Relationships with the family	
Improved	41 (22)
Because of a greater sharing	82 (18)
Because of a greater serenity	18 (4)
Worsened	19 (10)
Because of a to a difficulty of daily management	60 (6)
Because of discussions	40 (4)
Remained the same	40 (21)
Because they were already good before the lockdown	52 (11)
Because there have been no changes	29 (6)
Because the same problems/discussions occurred	19 (4)
Job conditions	
Still working	34 (18)
On site	72 (13)
Smart working	28 (5)
Activities in the house^*^	
Taking care of the house (e.g. cleaning, cooking)	68 (24)
Taking care of a family member (e.g. children, parents)	14 (5)
Reading or studying	6 (2)
Resting	6 (2)
Playing sports or hobbies	6 (2)
Emotional state	
General positive	45 (24)
General negative	51 (27)
Swinging	4 (2)
Change of the emotional state from before the lockdown	
Improved	25 (13)
Worsened	40 (21)
Remained the same	35 (18)

* *The answer categories for this question are not mutually exclusive*.

Referring to feelings about the closure of gambling opportunities due to COVID-19, 60% of AFMs reported being relieved, because their relative in treatment for GD had stopped gambling, while 13% reported being worried, because of the patient's online gambling (71%) or because they were afraid of future reopening (29%). Twenty-six percent of AFMs reported being indifferent because of the online gambling of their relative (36%) or because they had not thought about it (64%). During the previous month, AFM participants were involved in potentially addictive behaviours: 87% of them watched TV, 83% used their mobile phone, and 77% surfed the Internet. To a lesser degree, 38% smoked, 33% drank alcohol, 26% did online shopping, 19% played videogames, and 19% used medical substances. In most cases, participants were involved in these behaviours with the same frequency as before lockdown, except for an increase in the use of smartphones (30%), television (32%), and Internet (36%). Concerning psychological distress symptoms experienced during the previous month investigated through the SRT, the AFM participants mostly declared having been tired, worried, and apprehensive. The AFM participants reported that the symptom frequency was equal or greater than before lockdown (Table 4).

**Table 4:** Prevalence of physical and psychological symptoms of the SRT and perception about their changes from pre-lockdown period among the disordered gamblers' affected family members.

**Symptoms**	Prevalence^*^	**Perception of changes from**		
		**pre-lockdown period**		
		**Less than** **before**	**Equal**	**More than** **before**
Tired	74%	13%	34%	53%
Worried	74%	13%	72%	15%
Apprehensive	72%	6%	68%	26%
Afraid	66%	15%	70%	15%
Nervous	66%	15%	76%	9%
Guilty	62%	15%	47%	38%
Sad	62%	11%	70%	19%
Irritable	61%	15%	62%	23%
Difficulty in clearing the mind	57%	17%	64%	19%
Early awakenings	49%	9%	61%	30%
Circles in the head	47%	11%	47%	42%
Restless	43%	6%	83%	11%
Difficulty in falling asleep	43%	11%	62%	27%
Rapid heartbeat/palpitations	41%	7%	76%	17%
Loss of interest in the things to do	40%	15%	55%	30%
Difficulty in concentrating	40%	7%	76%	17%
Hopeless	34%	7%	74%	19%
Memory loss	34%	4%	81%	15%
Panic attacks	32%	9%	74%	17%
Feeling judged by others	32%	15%	77%	8%
Difficulty in breathing	30%	12%	73%	15%
Feeling a failure	28%	17%	50%	33%
Feeling inferior to others	26%	13%	66%	21%
Difficulty in making decisions	23%	15%	71%	14%
Without appetite	21%	4%	75%	21%

* *The answer categories for this question are not mutually exclusive*.

#### Personal Relationship With the COVID-19 Disease and Related Restrictions

From sections Introduction and Materials and Methods of the interview (see Supplementary Material), it appeared that 4% of participants were sick due to COVID-19 and 6% went into preventive lockdown because of contact tracing. About 42% of participants knew someone—on average, three persons—who had been affected by the virus and 6% knew someone who had died from COVID-19—on average, one person. The strongest emotions elicited by the pandemic were fear (*M* = 5.56; *SD* = 2.98; range: 1.00–10.00), stress (*M* = 6.53; *SD* = 2.99; range: 0.00–4.00), and anxiety (*M* = 6.36; *SD* = 3.18; range: 1.00–10.00). Attitudes towards the government's restrictions were generally favourable as the average score (*M* = 27.23; *SD* = 5.69; range: 11.00–35.00) was higher than the theoretical mean of the response scale (i.e., 21.00). During the previous week, 74% of the participants had gone outside the home, mostly to do shopping (60%), to go to work (25%), or to go to the pharmacy (26%)—that is, to do permitted actions. Everyone had, on average, one reason to exit the home (*M* = 1.40; *SD* = 1.21).

#### Perception of the Addicted Relative's Gambling Behaviour, Coping Strategies, and Feelings Towards Gambling

According to the results from section Results of the interview (see Supplementary Material), most of the participants reported that their addicted relative did not have a gambling problem at the time and that he/she had not gambled in the previous month. However, they still experienced specific situations that occurred because of their loved one's disordered gambling, with mood swings and quarrels being experienced by the largest proportion of participants. For all of the situations described, participants reported that gamblers engaged in behaviours with the same frequency as before the lockdown (Table 5). AFMs also still engaged in coping strategies to manage their relative's gambling behaviour. Those most frequently implemented were “*Helping the gambler in managing his/her financial situation*” and “*To take care of him/her”* (Table 5). Generally, participants reported that they used these coping strategies less or with the same frequency as before the lockdown. As for the emotional state towards gambling, participants' responses were classified in different categories based on their reported words. Most AFMs declared having a general negative state towards gambling, as indicated by the word “*disgust*,” “*anger*,” and “*hate*” (Table 5).

**Table 5:** Perception of the family member's gambling behaviour, coping strategies and feelings towards gambling among the affected family members.

	**No** **% (*n*)**	**Yes** **% (*n*)**		**Don't know** **% (*n*)**
Perception of a gambling problem for the disordered family member during the lockdown	79 (42)	10 (5)		11 (6)
Perception of gambling behaviour by disordered family member in the previous month	81 (43)	4 (2)		15 (8)
Situations resulting from family member's disordered gambling in the previous month^*^			**% (** ***n*** **)**	
Mood swings			64 (34)	
Quarrels			51 (27)	
Stealing or borrowing money without returning it			15 (8)	
Any participation in family activities			21 (11)	
Threats			4 (2)	
Coping strategies implemented to manage gambling behaviour^*^			**% (** ***n*** **)**	
Helping the gambler in managing his/her financial situation			49 (26)	
Helping the gambler to take care of him/herself			47 (25)	
Talking about what could be done to reduce gambling behaviour			45 (24)	
Clarifying the contribution that is expected from him/her in the family			43 (23)	
Making it clear to the family member that reasons for his/her gambling behaviour will no longer be accepted			41 (22)	
Becoming moody or emotional towards the family member			38 (20)	
Clarifying that gambling behaviour causes discomfort to family members			38 (20)	
Monitoring gambler's every movement			28 (15)	
Searching for evidence of your family member's gambling behaviour			24 (13)	
Asking the gambler to promise not to gamble again			23 (12)	
Making threats to the gambler			23 (12)	
Starting a fight with the gambler			7 (4)	
**Emotional state towards gambling**			**% (** ***n*** **)**	
General negative state			68 (36)	
Indifference			15 (8)	
Being worried and afraid about gambling			13 (7)	
Feeling compassion for problem gamblers			4 (2)	

* *The answer categories for this question are not mutually exclusive*.

#### Expectations Towards the Future

From section Discussion of the interview (see Supplementary Material), related to expectations towards the future, it appears that positive thoughts (e.g., perceiving things starting to get better and beginning to see a new future) were widespread. There was, however, also considerable agreement in negative perceptions of the future, including being afraid of how their relative in treatment for GD would behave, being pessimistic about the future, and thinking the gambler would never change (Table 6). Referring to the predictions of what would happen once the COVID-19 restrictions were lifted, most participants were concerned that the gamblers would relapse into gambling behaviours. In reference to the reduction of gambling opportunities due to the COVID-19 restrictions, the majority of the participants reported feeling relieved because their relative had stopped gambling. However, many AFMs were indifferent to this situation or still felt worried because of the possibility of gambling online (Table 6).

**Table 6:** Expectations towards the future among the affected family members.

	**% (*n*)**
Positive thoughts towards the future^*^	
Perceiving that things start to get better	79 (42)
Beginning to see a new future	68 (36)
Beginning to recognise the gambler as the person they used to know	66 (35)
Seeing things positively	74 (39)
Thinking something good will happen for the gambler	51 (27)
Negative thoughts towards the future^*^	
Being afraid of how the family member will behave in the future	42 (22)
Being afraid that the family member does not take things seriously enough	57 (30)
Being pessimistic about the future	55 (29)
Thinking the gambler will never change	79 (42)
Worried that the gambler will start gambling again	49 (26)
Predictions of what will happen once the COVID-19 restrictions are lifted	
Concerned that gamblers will relapse into gambling behaviours	77 (41)
Worried about a worsening of the COVID-19' pandemic	10 (5)
Thinking that the situation will not change	13 (7)
Feelings about the reduction of gambling opportunities due to	
COVID-19 limitations	
Feeling relieved because their family member had stopped to gamble	60 (32)
Feeling worried about the possibility to gamble online	13 (7)
Indifference	27 (14)

* *The answer categories for this question are not mutually exclusive*.

### AFMs and Their Relative in Treatment for GD

Looking at the self-reported gambling behaviour of the disordered gamblers, only 7% gambled during the lockdown: two on traditional/instant lotteries and one on online games, all less than once a week. Regarding gambling problem severity, the mean score on the SOGS was very low (*M* = 1.02, *SD* = 1.44, range: 0.00–8.00). As for the emotional state towards gambling, 38% of the responses reflected a generally negative state, as evidenced by the terms “*disgust*,” “*anger*,” and “*hate*,” while 48% reported being indifferent to gambling. On the other hand, 9% reported feeling guilty for gambling in the past, and 5% specified being worried about a possible relapse.

#### Quality of Family Relationships

Concerning familial relationships during the lockdown period, AFMs considered them as quite good, as did their relatives in treatment for GD. Indeed, through paired *t*-tests, we found a substantial equivalence [*t*_(35)_ = −0.15, *p* = 0.443, Cohen's *d* = 0.02] between the AFMs and the relative with GD in terms of the perception of family relationships.

When asked if the familial relationships changed from before the pandemic, no difference was detected between AFMs and the relative with GD [χ^2^(4) = 3.23, *p* = 0.261, ϕ = 0.30] when comparing the prevalence of responses indicating an improvement, a worsening, or a stability of the relationships. In detail, most AFMs reported that the quality of family relationships improved from before the lockdown or remained stable, as occurred among the group of relatives with GD. Among AFMs, those who reported that familial relationships improved during the lockdown period mainly attributed it to a greater family sharing, as evidenced by statements like “*we do more things together, we share some activities*,” and greater serenity, as evidenced by statements like “*we don't fight anymore, we have fun together*,” while those who reported a worsening of family relationships during the lockdown generally attributed it to daily management difficulties (i.e., “*always closed in the house, we were not happy*”) and to arguments and quarrels with other family members (i.e., “*we had some discussions*”). The AFMs who perceived a stability in their family relationships attributed this perception to the fact that there were no changes (i.e., “*nothing has changed*”), they were already good before (i.e., “*we had a good relationship before*”), or that the same problems/discussions always occurred (i.e., “*the same discussions always occurred*”). The relatives in treatment for GD who reported that family relationships improved during the lockdown period attributed this change to greater family sharing and greater serenity, and to a reduction of personal gambling behaviour (i.e., “*we don't fight because I don't gamble anymore*”). The gamblers who reported a worsening of family relationships during the lockdown generally attributed it to the restrictions related to COVID-19 (i.e., “*it was difficult to spend a lot of time together in the house*”) and to arguments and quarrels with other family members (i.e., “*there have been moments of nervousness*”*)*. Those who declared no changes generally reported that the situation remained stable because there were no changes or because they were already good before (i.e., “*nothing has changed, our relations were already good*”) (Table 7).

**Table 7:** Perception of familial relationships among the affected family members and the relatives in-treatment for gambling disorders.

	**Affected**			**Relatives**		
	**family members**			**in treatment for GD**		
	**M**	**SD**	**range**	**M**	**SD**	**range**
Perception of familial relationships during the lockdown	7.89	1.57	4.00–10.00	7.92	1.54	5.00–10.00
	**Response**		**% (** ***n*** **)**	**Response**		**% (** ***n*** **)**
Perceived change of the familial relationships with respect to before the lockdown	Improved		41 (17)	Improved		59 (25)
	Worsened		14 (6)	Worsened		11 (4)
	Stable		45 (19)	Stable		30 (13)
Emotional state during the lockdown	Positive		40 (17)	Positive		64 (27)
	Negative		55 (23)	Negative		29 (12)
	Swinging		5 (2)	Swinging		7 (3)
Perceived change of the emotional state with respect to before the lockdown	Improved		22 (9)	Improved		31 (13)
	Worsened		45 (19)	Worsened		36 (15)
	Stable		33 (14)	Stable		33 (14)

#### Emotional State

Concerning emotional state during the lockdown period, statistical comparisons indicate a significant difference characterised by a moderate effect size between the AFMs and the relatives with GD [χ^2^(4) = 8.39, *p* = 0.039, ϕ = 0.45]. The emotional state of the AFMs was more negative than in the gamblers. While most of the AFMs' responses reflected a generally negative state (“*tired, bored, depressed, sad, worried”*), among their relatives in treatment for GD, the majority of the responses reflected a generally positive state, as evidenced by terms like “*well, better, calm, serene, happy*” (Table 7).

In reference to emotional state compared to before the lockdown, a significant difference, characterised by a moderate effect size, was evidenced between the AFMs and the relatives with GD [χ^2^(4) = 8.50, *p* = 0.038, ϕ = 0.45]. The emotional state of the AFMs compared to before the lockdown was more negative than in the gamblers. Among AFMs, most of the participants reported that their emotional state worsened, but among their addicted relatives, there was a substantial equal distribution of the responses across improved, worsened, and remained stable (Table 7). Both among the AFMs and their addicted relatives who reported an improvement in their emotional state during the lockdown, the majority attributed the change to greater family sharing and greater serenity (i.e., “*family tensions have decreased, there is more time to be with family members*”). The gamblers also cited the reduction of personal gambling behaviour (i.e., “*I don't play anymore and I'm enjoying life more*”). Among the AFMs who reported a worsening of their emotional state during the lockdown, most of them attributed it to daily management difficulties (i.e., “*responsibilities at home have increased, being at home I have to take care of more things*”), while among their relatives in treatment for GD, the majority attributed the worsening to COVID-19 restrictions or to concerns for work and future (i.e., “*many freedoms have been taken away from me, I am worried about the future*”). Both among the AFMs and their gambling relatives who reported that their emotional state remained stable, the majority reported that there were no changes (i.e., “*nothing has changed, I had already found a balance*”) (Table 7).

## Discussion

The general goal of the study was to assess the psychological state of the AFMs of disordered gamblers during the COVID-19-related lockdown in Italy when most land-based gambling offerings were unavailable. Indeed, although some studies have been conducted on gambling behaviour in the general population or in clinical samples, little attention has been focused on disordered gamblers' AFMs. To better understand the specificity of their status, we also analysed AFMs in comparison to their relatives in treatment for GD. The relative majority of AFMs indicated a general positive emotional state, perhaps because of the large declared sense of relief due to the closure of gambling opportunities that led their addicted relatives to stop gambling, and a general perception of good family relationships that are perceived as improved from before the lockdown. This can be attributed to greater family sharing and to the lower work-related stress that may have been generated by the lockdown. However, the majority of AFMs reported that their emotional state was worse than or equal to before the lockdown, rather than improved.

Despite the substantial absence of gambling behaviour and the specific addiction symptoms, the AFMs appear to still perceive problems in the family due to their relative's gambling addiction, such as mood swings, quarrels, and cases of stealing/borrowing money, that were also present before the pandemic. At the same time, the AFMs are still engaged in coping strategies to help their disordered gambler in managing financial problems or in reducing his/her gambling behaviour. AFMs were also largely involved in potential addictive behaviours, especially watching TV, spending time on the mobile phone, and using Internet, and reported a greater frequency of these behaviours than before the lockdown. However, these data must be considered in light of the fact that during the COVID-19 lockdown, a high frequency of addictive behaviours was also detected in the Italian general population (e.g., Panno et al., 2020; La Rosa et al., 2021).

Importantly, AFMs experienced a large amount of the physical and psychological symptoms of distress, being tired, worried, and apprehensive, and perceived the symptomatology equally or to a greater extent than before the lockdown. They also perceived more fear, stress, and anxiety than before the lockdown. This stress could be relatively linked to the COVID-19 threat as almost none of the AFMs became ill because of the SARS-CoV-2 virus or knew someone affected or died of this reason. In addition, they had a positive attitude towards the normative rules to fight against the widespread pandemic.

It is important to note that despite the cessation of gambling, AFMs still perceived some problematic behaviours from their addicted relatives, and they still implemented coping strategies aimed at managing the gambling problem. Even if they had a generally positive orientation towards the future, they expressed fear when thinking about the reopening of gambling opportunities at the end of the lockdown.

When analysing AFMs with their matched disordered gamblers, both groups of participants reported good family relationships and, generally, perceived them as improved from before the lockdown. However, significant differences, characterised by moderate effect sizes, were evidenced when looking at general emotional state during the lockdown and the perception of worsening/improvement/stability of the emotional state from before lockdown. AFMs were more prone to perceive a general negative emotional state and a worsening from before the lockdown as compared to their relatives with GD. However, the comparisons must be read in light of a small sample size for the two groups and taking into account that while the patients were tested in April–May 2020, the AFMs were interviewed in May–June 2020. Although gambling behaviour restrictions remained the same during these two periods, the incomplete overlap of the time administrations across the two groups of participants complicates direct comparison.

This study offers many insights because the scenario generated by the COVID-19 lockdown allowed a sort of natural experimental verification of many data that were not otherwise assessable. First, the severity of emotional and health harms experienced by disordered gamblers' family members does not seem to be very much relieved as a consequence of lower pressure due to the reduced exposure to gambling offerings and the triggers of their loved ones. They also do not seem to feel better because their gambling relatives stopped gambling. In other words, the AFMs' psychological malaise does not end. It does not automatically alleviate itself with the cessation of gambling behaviour and/or symptoms of their addicted relative and does not end as a result of the absence of gambling opportunities.

This study reveals symptoms compatible with real post-traumatic stress disorder (PTSD) in AFMs, characterised by experiencing ongoing distress in the present and anticipating possible bad scenarios for the future, quite similar to intrusive thoughts that are elicited by foreshadowing what would happen after the lockdown, namely, the certainty of the reopening of land-based gambling opportunities. The closure was only transitory, just a parenthesis in a wider situation that arouses apprehensions and concerns for the AFMs. The cessation of the noxious stimulus seems not to be enough to mitigate their stress and strain. The damage suffered as a result of living with a disordered gambler seems to still be present and disturbing even when most of land-based gambling activities had closed and their relative's gambling behaviour has ceased. These considerations suggest that the cessation of the noxious stimulus is not sufficient to attenuate their previous trauma. Family members went through real traumatic events in their lives while dealing with the GD of a relative, and for this reason, they require a clinical rehabilitation work specifically centred on processing and overcoming trauma. Our observation about the presence of symptoms compatible with PTSD among the AFMs follows the clinical interpretation of McComb et al. (2009). Indeed, by comparing findings from the sexual addiction literature with the impact of problem gambling on affected families, they observed that both disorders involve a similar pattern of hiding behaviours that lead spouses to experience symptoms of PTSD, either following the sudden discovery of a sexual addiction or the sudden disclosure of a gambling problem. They noted that the early warning signs of gambling problem frequently go unrecognised until there are devastating consequences for a family. Because of the secrecy often associated with gambling, they underline how the disclosure of a gambling problem is often sudden, drastic, and devastating. Hence, it is often described as a “traumatic experience” for the family.

In light of our results, in analogy to the well-known concept of increased harm and risks deriving from exposure to passive smoking, i.e., “environmental tobacco smoking” (Eriksen et al., 1988), we are proposing the analogous expression of “passive gambling.” Being exposed to someone else's gambling excessive practise configures an important psycho-socio-health risk and threat to the well-being for the significant others involved because of their proximity. The effect of this prolonged and often chronic exposure remains, to date, under-researched. Related health hazards are therefore underestimated, both in terms of the impact on public and private health and in terms of the size of the population affected. This study thus points out how *passive gambling* is an important risk factor for the well-being of disordered gamblers' significant others and strongly suggests that, in countries such as Italy where legalised gambling is authorised, it should become a major public health concern, as the AFMs of addictive gamblers are a large vulnerable group that deserve more research, prevention, and intervention efforts.

Our findings reinforce what many researchers have pointed out: awareness should be raised at a global level about the needs of families affected by addiction (Velleman et al., 2015; Klevan et al., 2019). They suggest disseminating a non-pathological and family member-centred model of the circumstances and needs of family members affected by their relatives' addictions to promote research about the experiences of family members affected by their relatives' addictions around the world; to promote good, evidence-based prevention and treatment practise relevant to the needs of affected family members; and to advocate with policymakers, including international organisations and national governments, for greater awareness of the circumstances and needs of family members affected by their relatives' addictions and for better support services for the AFMs. Of course, this implies a more general reconsideration of the entire offer of gambling in the states that allow it (Orford, 2020).

As far as future research, it is necessary to reinforce the aspects of assessment. More sophisticated and more targeted tools are needed for this specific group, and above all, tools adapted in Italian. Regarding the clinical interventions for AFMs, it is necessary to offer focused treatments for the AFMs and specific support for their risk of developing chronic stress, strain, hopelessness, and erroneous thoughts and negative metacognitions about others (mistrust, fear of betrayal, etc.) built on the traumatic experience with their disordered gambler relative. Indeed, the achievement of these goals also depends on early recognition and legitimation of their request for help. Particularly, not only taking charge and offering consultancy of AFMs in parallel or together with their gambler relatives (individually, in couples or in groups), as is already sometimes the case, but thinking about an early takeover of the AFMs' own support needs and guaranteeing them qualified targeted responses (Rodda et al., 2019). According to our study, it is important to consider AFMs not only as “co-therapists at home” or “guardians of the gambler” (i.e., only as a potential positive resource to support the gambler patient), but also welcoming them as suffering people in need of care for their own health needs. The preventive and clinical attention to the AFMs' psychological state must also be read considering the risk of a “vicious cycle” between the family member and the disordered gambler. Indeed, a negative psychological state for the AFM may prove a trigger for problem gamblers to relapse into their problem gambling, thereby creating a vicious cycle that unfortunately reflects negatively on their own well-being as well. Finally, under pandemic conditions, there is an extra need to identify methods for promoting more successful adherence to behavioural advice and support strategies aimed at vulnerable groups, such as AFMs, to improve their coping strategies and offer preventive interventions that can be delivered to reduce their mental health threats (which in a period of generalised stress can be even more demanding for them), while minimising distress and promoting well-being.

This study has some limitations. The sample size was small, and the data were collected only in northern Italy. In addition, the AFMs interviewed were mainly female. These data are representative of the targeted population as the majority of disordered gamblers are, according to Mastrobattista et al. (2019), males and their caregivers are mostly women. Nevertheless, the differences found may have been affected by gender differences as women tend to experience higher levels of distress than men (e.g., Matud, 2004). This aspect would benefit from being investigated with further specific research in order to discriminate between alternative explanatory hypotheses, i.e., intense suffering linked either to being female or being gamblers' AFMs or both of the conditions. Moreover, the dichotomization of the scores on the quantitative measures at the item level, despite being functional to have an immediate report of the prevalence of each behaviour/symptom analysed, does not allow direct comparisons with previous studies using the same scales (e.g., Arcidiacono et al., 2010). However, the cited studies used the measures employed in this sample with AFMs regarding other addictions and not gambling. Another limitation is that it is not possible to attribute the stress experienced by the AFMs to the problem gambler because the effects could instead be due to the stresses of the pandemic or an interaction between the gambling problem and the pandemic, and because neither pre-pandemic nor mid-pandemic data were collected on the same stress measure in the general Italian population against which the AFM data can be compared. Nevertheless, considering AFMs as an at-risk and vulnerable group because of their enduring encounter with disordered gambling harms, the pandemic might have only worsened their malaise. Therefore, further specific research is needed.

Despite the limitations, the study also has strengths, as it was conducted both in public and private addiction services, and it was possible to match the responses of 42 AFMs with those of their addicted relatives. Finally, this study focused on a neglected and suffering target, the AFMs, and highlighted the traumatic nature of the experience of living with someone else's gambling addiction. This is particularly important considering that people who find themselves in this little-known situation represent a large slice of the population whose physical and mental health is compromised by indirectly experiencing the pathology of gambling addiction. In addition, the study carried out highlights this situation. Deepening the comparison between AFMs and their disordered gambler relatives with homogeneous and more sophisticated tools, repeating the study with larger samples, analysing the role of the type of kinship, and verifying the trend of the situation in the post-COVID-19 period are recommendations for future development to this research concern.

## Data Availability Statement

The raw data supporting the conclusions of this article will be made available by the authors, without undue reservation.

## Ethics Statement

Ethical review and approval was not required for the study on human participants in accordance with the local legislation and institutional requirements. The patients/participants provided their written informed consent to participate in this study.

## Author Contributions

DC, MD, RS, and CP: conceptualisation. MD, DC, CP, CB, and RS: methodology. CB, MD, and CP: formal analysis. EQ, AC, and RS: investigation. MD and CB: data curation. DC, MD, CB, and RS: writing—original draft preparation, and writing—review and editing. CP: supervision, writing—review and editing. All authors have read and agreed to the published version of the manuscript.

## Conflict of Interest

The authors declare that the research was conducted in the absence of any commercial or financial relationships that could be construed as a potential conflict of interest.

## Publisher's Note

All claims expressed in this article are solely those of the authors and do not necessarily represent those of their affiliated organizations, or those of the publisher, the editors and the reviewers. Any product that may be evaluated in this article, or claim that may be made by its manufacturer, is not guaranteed or endorsed by the publisher.
