# Supplementary material for: What About Affected Family Members of Disordered Gamblers During the COVID-19 Pandemic? A Study in Italy During the Lockdown Restrictions
Source: Front Psychol. 2022 Apr 26;13:801835. doi: 10.3389/fpsyg.2022.801835 (PMC9088675; doi:10.3389/fpsyg.2022.801835)
Supplement: Supplementary file 1 [file Table_1.docx]

**SUPPLEMENTARY MATERIAL**

**Questions and response options of the telephone interview to the disordered gamblers’ Affected Family Members (AFMs)**

| **Section** | **Variable** | **Questions and response options** |
| --- | --- | --- |
| 1 | Home environment | “How many square meters is the house where you live?” [open question].  “Are there open spaces in the house?” [*yes*, *no*]; “If yes, which of the following? [*garden, terrace, vegetable garden*].  “How many people are you living with?” [open question]. |
|  | Familiar relationships | “Please rate the relationships with your family members” [from a minimum of *1* – *worst*, to a maximum of *10* – *excellent*].  “In reference to this lockdown period, do you think the relations with the family members are…?” [*improved*, *worsened*, *remain the same*]; “Why?” [open question]. |
|  | Job conditions | “Which of the following is your working condition at the moment?” [*job interruption, work on-site, smart working*]. |
|  | Emotional state | “How have you been feeling over the last few weeks?” [open question].  “How do you feel thinking that the gambling opportunities were closed?” [*relieved, indifferent, worried anyway*]; “Why?” [open question]. |
|  | Perceptions about changes from the pre-lockdown period to the ongoing lockdown period with respect to the emotional state | “With respect to the period prior to the COVID-19 pandemic, how do you feel?” [*better, worse, the same*]; “Why?” [open question]. |
|  |  |  |
|  | Other potentially addictive behaviours | “How frequently did you have the following behaviours during the last month? Alcohol use, videogame playing, Internet use, smoking, substance use, online shopping, TV watching, and smartphone use” [*never*, *sometimes*, *often].* |
|  | Other potentially addictive behaviours from the pre-lockdown period to the ongoing lockdown period | “With respect to the period before the beginning of the lockdown, have you engaged in the following behaviours more or less frequently? Alcohol use, videogame playing, Internet use, smoking, substance use, online shopping, TV watching, and smartphone use?” [*less than before*, *equal, more than before*]. |
|  | Psychological distress | *Symptom Rating Test* (SRT; Kellner & Sheffield, 1973; Italian version: Fava et al., 1983) |
| 2 | Personal impact with the COVID-19 disease | “Have you tested positive for COVID-19? [*yes*, *no*].  “Have you been in quarantine due to contact tracing?” [*yes*, *no*].  “Do you know people affected by COVID-19?” [*yes*, *no*].  “Do you know people who have died because of COVID-19?” [*yes*, *no*]. |
|  | Perceived emotional impact of the COVID-19 pandemic | “Please rate how you felt, from a minimum of 1 (*very little*) to a maximum of 10 (*very much*), regarding each of the following emotional states: anxiety; depression; fear; stress; and anger”.  “Please rate your experience of eating and sleeping problems, from a minimum of 1 (*very little*) to a maximum of 10 (*very much*)”. |
|  | Perception of the adequacy of the national restrictions | “Please report the extent to which you agree with the following statements using a 5-point Likert scale from 1 (*totally false*) to 5 (*totally true*)”. |
|  | Behaviour adhered to the rules | “How frequently did you leave home in the last week?” [*never*, *1–3 times*, *4–6 times*, *7–9 times*, *more than 10 times]*.  “For which of the following reasons?” [*walking alone*; *walking with someone in my family*; *walking with friends*; *returning to the own residence*; *going to work*; *doing the shopping*; *running urgent errands*; *going to the pharmacy;* *going to the hospital*; *going to the doctor*; *assisting relatives/friends in need].* |
| 3 | Perception of a gambling problem for the family member who gambles | “Please report if your family member who gambles excessively:   - *Has a gambling-related problem;* - *Does not have a gambling-related problem;* - *I don’t know.”* |
|  | Perception of gambling behaviour concerning the family member who gambled during the last month | “Has your family member who gambles excessively gambled in the last month?”  [*yes*, *no*, *I don’t know*] |
|  | Situations that occurred in the family as a result of the family member’ problem gambling | “To your knowledge, have any of the following situations occurred in the past month as a result of your family member’s problem gambling?”  [*not at all*, *once/twice*, *sometimes*, *often*, *I don’t know*]   - *Mood swings* - *Steals or borrows money and does not pay it back* - *Quarrels* - *Threats* - *Participation in family activities fails* |
|  | Changes in the frequency of the situations that occurred in the family as a result of the family member’s problem gambling from the pre-lockdown period to the ongoing lockdown period | “With respect to the period before the beginning of the lockdown, how frequently has your family member who gambles exhibited the following behaviours during the last month?”  [*less than before*, *equal,* *more than before*] |
|  | Coping strategies used to manage the family member’s problem gambling | “During the last month, how frequently have you engaged in the following behaviours because of your family member’s problem gambling?  [*never*, *once/twice*, *sometimes*, *often*]   - Did you talk frankly with your family member about what could be done about his/her gambling behaviour? - Have you started an argument with your family member about his/her gambling habits? - Have you made it clear to your family member that his/her gambling behaviour causes you discomfort, and that he/she needs to change? - Have you invited your family member to swear or promise not to gamble again? - Did you feel too short of hope to do anything? - Have you become moody or emotional towards your family member? - Did you monitor every movement of your family member or watch over him/her? - Did you made it clear to your family member that you will no longer accept reasons for his/her gambling behaviour, and that you will no longer cover it? - Did you make threats you didn’t really plan to put into practice? - Did you make it clear to your family member what you expect from him/her to make his/her own contribution to the family? - Did you stop to talk to his/her family member to help him/her manage his/her financial situation? - Have you sought evidence of your family member’s gambling behaviour? - Have you ever thought above all of yourself, by taking care of you or by indulging in something pleasant?” |
|  | Changes in the coping strategies used to manage the family member’s problem gambling from the pre-lockdown period to the ongoing lockdown period | “With respect to the period before the beginning of the lockdown, how frequently have you engaged in the following behaviours because of your family member’s gambling behaviour?”  [*less than before*, *equal, more than before*] |
|  | Emotional state toward gambling | “What do you feel about gambling at this moment?” [open question]. |
| 4 | Expectations toward the future | “For each of the following sentences, please indicate how much you agree [*very much agree*, *quite agree*, *I don’t know*, *quite disagree*, *very much disagree*]   - I am beginning to foresee a new future - I’m scared about how my family member will fare in the future - My family member doesn’t take things seriously enough - I am pessimistic about the immediate future - I believe that something very positive will come out of all this for my family member - Nothing will ever change, my family member has no way out - I am concerned that my family member will continue to gamble until the end - Things are beginning to improve - I am beginning to recognize him/her as the person I knew - Now I see things more positively” |
|  | Personal predictions concerning what will happens once the COVID-19 related restrictions will be loose | “What do you think will be happen once the COVID-19 related restrictions have been eased?” [open question]. |
|  | Feelings about the closure of gambling opportunities due to COVID-19 | “How do you feel when thinking that gambling opportunities are not available?”  [*relieved*, *worry*, *indifferent*] |

*Note*: The underlined questions were also included in the telephone interview for the family member under treatment for GD.
